# Generative processing underlies the mutual enhancement of arithmetic fluency and math-grounding number sense

**DOI:** 10.3389/fpsyg.2014.01326

**Published:** 2014-11-19

**Authors:** Ivilin P. Stoianov

**Affiliations:** ^1^Laboratoire de Psychologie Cognitive, Centre National de la Recherche Scientifique and Université d'Aix-MarseilleMarseille, France; ^2^National Research Council of Italy, CNR, Goal-Oriented Agents Lab, Institute of Cognitive Sciences and TechnologiesRome, Italy

**Keywords:** cognitive modeling, generative model, predictive coding, numerical cognition, approximate number system, arithmetic fluency, symbol grounding

Number skills are popularly bound to arithmetic knowledge in its symbolic form, such as “*five* + *nine* = *fourteen*,” but mounting evidence suggests that these symbolic relations are actually grounded, i.e., computed (see Harnad, 1990) on noisy internal magnitude representations that bear our general understanding of numbers and further improve with math experience (Figure 1). Multiple lines of evidence support the idea of semantics-based arithmetic, including behavioral research on humans (Gallistel and Gelman, 1992), animals (Gallistel and Gelman, 2000; Rugani et al., 2009), development (Halberda et al., 2008), mathematical disability, i.e., dyscalculia (Butterworth, 1999; review, Butterworth et al., 2011), and computational modeling (Stoianov et al., 2004; review, Zorzi et al., 2005). Even more intimate relation between the number skills and the internal noisy magnitudes was recently demonstrated in several studies showing finer magnitude representations in subjects with greater arithmetic fluency (e.g., Nys et al., 2013; Piazza et al., 2013), also caused by extensive math studying during higher education (Lindskog et al., 2014). Here we discuss how these findings could be explained within a generative framework of cognition, according to which top-down predictive connections play a key role in the computing of low- to high-level representations (e.g., Friston, 2010; Clark, 2013).

**Figure 1:**
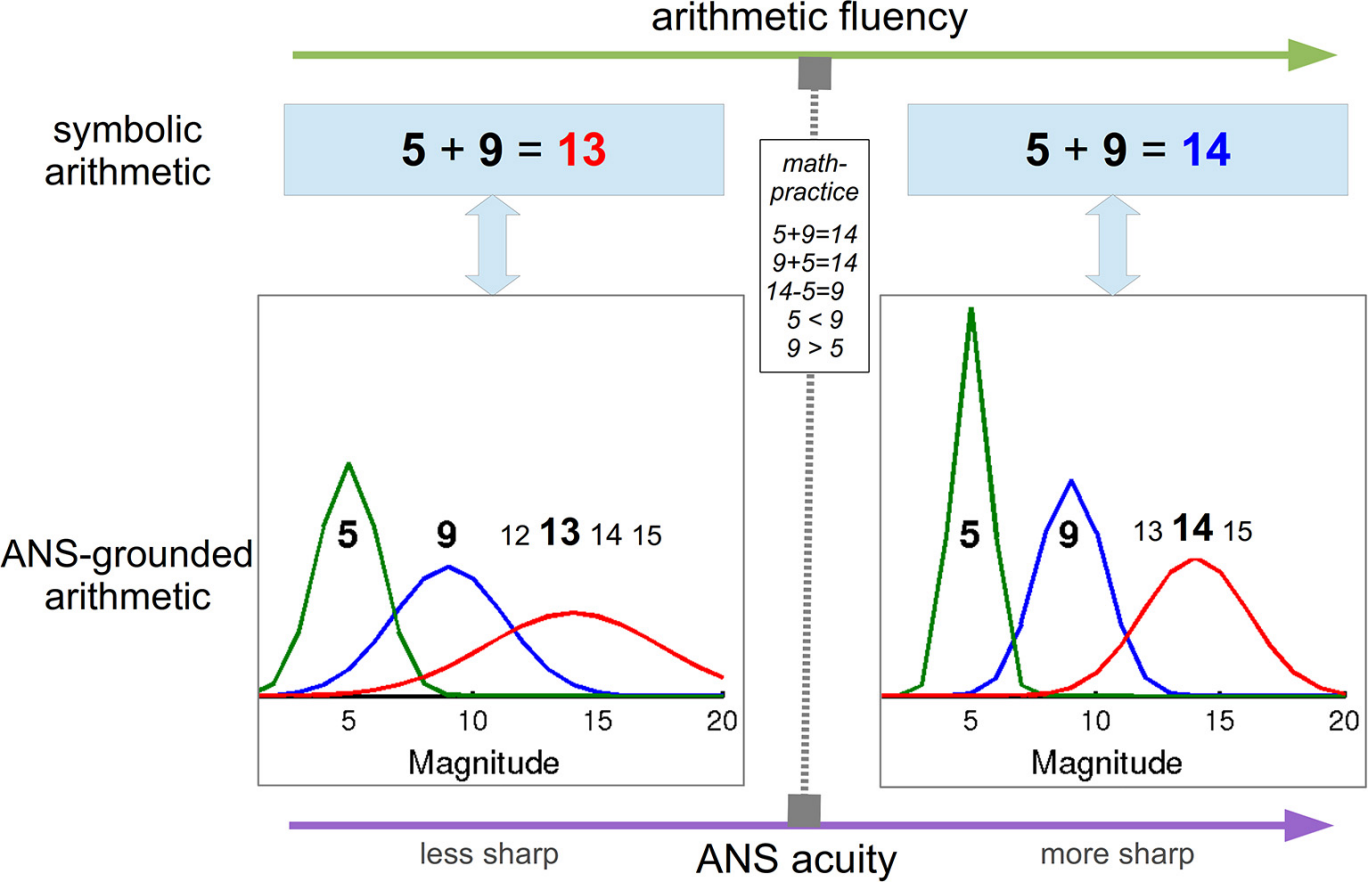
**ANS-grounding of math skills provides a link between the levels of arithmetic fluency and ANS acuity**. Less noisy (i.e., sharper) ANS supports more accurate associative arithmetic retrieval. In turn, math practice, especially explicit or implicit magnitude discrimination tasks not only improves the arithmetic skills, but also sharpens the discrimination process and the underlying noisy magnitudes by means of top-down generative processing.

The noisy internal magnitude representations also known as Approximate Number System (ANS), or Number Sense are systematically found in the intraparietal silcus and prefrontal cortices (Dehaene, 1997; Viswanathan and Nieder, 2013) and one principle method to investigate them is to characterize the ability to quickly and approximately estimate the number of objects seen (Jevons, 1871). This phylogenetic ability is qualified as a visual sense (Burr and Ross, 2008), the mechanism of which emerged in generative neural networks that learn to efficiently encode visual numerosities (Stoianov and Zorzi, 2012).

One crucial property of the internal magnitude representations is their systematically increasing imprecision (Figure 1; Gallistel and Gelman, 2000; Dehaene, 2003) characterized by a subject-specific constant known as ANS acuity (Halberda et al., 2008), which at the behavioral level is associated with log-linear performance decrement as the magnitude increases. In numerosity comparison, the probability to select the greater numerosity is a sigmoid function of the log-ratio of the compared magnitudes that is characterized by a discriminability (Weber) fraction *w* describing the slope of the sigmoid, whereby the better the discriminability, the closer is the sigmoid to a step-function, for which *w* = 0 (Piazza et al., 2013; Cappelletti et al., 2014). The behavioral discriminability coefficient *w* is closely related to the internal ANS acuity (Piazza et al., 2013). The ANS acuity progressively improves along with development, with corresponding *w* = 1 in the first few months of life to about *w* = 0.24 in healthy adults (Piazza et al., 2010), to worsen then with ageing to more than *w* = 0.30 (Cappelletti et al., 2014).

The intriguing question we explore here is whether ANS improves along with refinement of the mathematical knowledge it supports, that is, whether math-studying improves general quantity understanding. A hint about this was provided by a study on a curious Amazonian Mundurucù population with two levels of math education (Piazza et al., 2013). The effect of math-studying on ANS acuity, controlling for age, was impressive: *w* = 0.31 for adults that had never studied math and *w* = 0.19 for math-educated adults. Sure, this is a study on a particular population, with an educational system that permitted to find subjects allowing the dissociation between age and education (see also Nys et al., 2013), and it remained unclear whether prolonged math schooling in cultures with broad educational system is associated with further improvement of the ANS.

Lindskog et al. (2014) investigated this issue with first- and third-year university students majoring in disciplines with various levels of initial math-expertise and amount of math-studying, ranked in the order: humanity-disciplines, with expected basic math-background, and no math-studying, business-disciplines with average math-background and applied math-studying, and math-disciplines with high math proficiency and mostly theoretical math-studying. The ANS-acuity of the students was evaluated using visual numerosity comparison tasks and supplementary assessment measured their arithmetic fluency. Overall, the arithmetic fluency increased along with the rank of the math-expertise and similarly, the ANS-acuity tended to improve along with this rank, from about *w* = 0.27 in humanity disciplines to about *w* = 0.24 in math disciplines. Moreover, a regression analysis showed that the two measures were correlated even after controlling for possible confounds. Note that this analysis could not reveal causality, but it is legitimate to assume that different levels of past mathematical experience bring to different levels of math-fluency and as the link shows, to corresponding levels of ANS-acuity.

Indeed, the arithmetic fluency improved in the third-year business- and math-students relative to their first-year pairs, confirming the expected causal role of math-training. However, only the students with an intermediate-level of math expertise obtained apparent ANS benefit from the two more years of math studying, with *w* = 0.27 of the first-year business students and *w* = 0.23 of the third-year students (Figure 1C of Lindskog et al., 2014). The ANS acuity of all math-students was almost as good as that of the third-year business-students, which altogether was significantly better than the ANS-acuity of the first-year business students and all humanity-studying subjects that did not study math. Math-students probably already had their ANS-acuity at ceiling, or their advanced math training was maybe too-abstract to affect the ANS. The math-training of the business students likely involved concrete calculi that not only improved their arithmetic fluency, but also their ANS.

Curiously, the link between the ANS acuity and arithmetic fluency dissociated two apparently very similar methods for ANS-acuity assessment: one based on the comparison of two simultaneously presented numerosities and another one based on their sequential presentation (see also Lindskog et al., 2013). Both methods resulted in close measures but only the first one correlated with arithmetic fluency. The suspicion was that mediating short-term memory in the sequential method introduced variability that masked the link, but further research is needed to clarify this potentially informative issue. Clearly, the interesting results from the study by Lindskog et al. (2014) prompts for even more direct demonstration of the causal link between arithmetic studying and quality of the grounding ANS. Longitudinal study with within-subject design could decrease the experimental noise; measures of initial mathematical expertise and quantification of arithmetic exercising could allow objective analyses, and better control of numerosity stimuli could provide further insights (e.g., Cappelletti et al., 2014).

The critical question then regards the mechanism underlying the observed ANS improvement along with greater arithmetic precision and math experience. Here we provide a tentative proposal that radically differs from symbol-based accounts according to which experience with symbolic numerals essentially tightens the borders between the underlying noisy magnitudes (e.g., Nys et al., 2013). First, in a series of model-based studies we showed that principle behavioral signatures of simple arithmetic can be replicated only when semantic number coding is included (Stoianov et al., 2002, 2003). Most instructive was the functional specialization found in a fully-connected associative connectionist model that included both symbolic and semantic components. When the model was enquired to retrieve facts with symbolic numerals as input, it first accessed their semantic representations; then it virtually “calculated” the semantic representation of the result in an emergent component building semantic arithmetic memory, and finally it activated the corresponding symbolic numeral. The critical role of the semantic component was corroborated in simulations of dyscalculia in which only lesions applied to the semantic component degraded the performance (Stoianov et al., 2004). Thus, learners equipped with symbolic (domain-general) and semantic (domain-specific) magnitude representations (i.e., ANS) exploit properties of the semantic codes to efficiently learn and perform arithmetics (Figure 1).

It is now apparent that improving the acuity of ANS would bring to more accurate semantic “calculations” and thus greater arithmetic fluency measurable at the symbolic level. Less intuitive is how further mathematical training could enhance the grounding ANS. We propose that the mechanism of ANS improvement is based on practicing number comparison tasks which, by “computing” upon the grounding ANS, gradually improve, on one hand the magnitude discrimination process and on the other, the precision, i.e., discriminability, of the noisy magnitudes. Deep top-down effects (e.g, on the magnitude representations) are natural in generative perceptual structures that operate, i.e., compute higher-level representations by constantly predicting the lower-level input through top-down generative connections and there is plenty of evidence that generative computations underly perception in the brain (e.g., Friston, 2010; Clark, 2013). A good starting point for a generative computational model that could simulate the effects of math studying on ANS could be the neurocomputational model of visual numerosity estimation by Stoianov and Zorzi (2012), on the top of which could be added math-operation (e.g., magnitude-comparison) modules (for insights, see Zorzi et al., 2013). Note that math studying, especially applied math, generally includes number-comparison tasks; arithmetic exercises should also be useful since arithmetic problem solving frequently relies on strategies based on operand ranking, i.e., discrimination (Butterworth et al., 2001; Campbell and Xue, 2001). Thus, math-studying, especially practicing calculi, should improve the acuity of the underlying ANS and the mathematical abilities it supports (Figure 1), including decisions on the size of visual numerosities, which task is typically used to assess the ANS acuity. Important corroboration for our proposal comes from novel experimentation with fish showing that numerosity discrimination based only on ANS improves following extensive training on this task (Bisazza et al., 2014).

In conclusion, converging evidence, including recent human data regarding higher education (Lindskog et al., 2014) imply that the arithmetic skills are not only grounded on noisy magnitudes but also causally affect the ANS along with math experience. Here we discussed supporting experimental data and outlined a tentative theoretical account of how ANS improvements allow better numerical skills and how in turn math studying could sharpen the ANS. The work might have implications regarding the math skills of healthy individuals and subjects with dyscalculia, providing theoretical basis for interventions (Butterworth et al., 2011). The result might also prompt relevant comparative animal research; we know for example that even chicken posses basic arithmetic knowledge (Rugani et al., 2009). The proposed theoretical account could be verified computationally using the generative model of Stoianov and Zorzi (2012).

## Acknowledgment

This research was supported by a Marie Curie Intra European Fellowship within the 7th European Community Framework Programme PIEF-GA-2013-622882 granted to Ivilin P. Stoianov

### Conflict of interest statement

The Associate Editor Anna M. Borghi declares that, despite being affiliated to the same institution as the author, the review process was handled objectively and no conflict of interest exists. The author declares that the research was conducted in the absence of any commercial or financial relationships that could be construed as a potential conflict of interest.

## References

[B1] Bisazza A. Agrillo C. Lucon-Xiccato T. (2014). Extensive training extends the numerical abilities of guppies. Anim. Cogn. 17, 1413–1419. 10.1007/s10071-014-0759-7 24859818

[B2] Burr D. Ross J. (2008). A visual sense of number. Curr. Biol. 18, 425–428. 10.1016/j.cub.2008.02.052 18342507

[B3] Butterworth B. (1999). The Mathematical Brain. London: McMillan.

[B4] Butterworth B. Varma S. Laurillard D. (2011). Dyscalculia: from brain to education. Science 332, 1049–1053. 10.1126/science.1201536 21617068

[B5] Butterworth B. Zorzi M. Girelli L. (2001). Storage and retrieval of addition facts: the role of number comparison. Q. J. Exp. Psychol. 54A, 1005–1029. 10.1080/713756007 11765730

[B6] Campbell J. I. D. Xue Q. (2001). Cognitive arithmetic across cultures. J. Exp. Psychol. Gen. 130, 299–315. 10.1037/0096-3445.130.2.299 11409105

[B7] Cappelletti M. Didino D. Stoianov I. Zorzi M. (2014). Number skills are maintained in healthy ageing. Cogn. Psychol. 69, 25–45. 10.1016/j.cogpsych.2013.11.004 24423632

[B8] Clark A. (2013). Whatever next? Predictive brains, situated agents, and the future of cognitive science. Behav. Brain Sci. 36, 181–204. 10.1017/S0140525X12000477 23663408

[B9] Dehaene S. (1997). The Number Sense. New York, NY: Oxford University Press.

[B10] Dehaene S. (2003). The neural basis of the Weber-Fechner law: a logarithmic mental number line. Trends Cogn. Sci. 7, 145–147. 10.1016/S1364-6613(03)00055-X 12691758

[B11] Friston K. (2010). The free-energy principle: a unified brain theory? Nat. Rev. Neurosci. 11, 127–138. 10.1038/nrn2787 20068583

[B12] Gallistel C. Gelman R. (1992). Preverbal and verbal counting and computation. Cognition 44, 43–74. 10.1016/0010-0277(92)90050-R 1511586

[B13] Gallistel C. Gelman R. (2000). Non-verbal numerical cognition: from reals to integers. Trends Cogn. Sci. 4, 59–65. 10.1016/S1364-6613(99)01424-2 10652523

[B14] Halberda J. Mazzocco M. M. M. Feigenson L. (2008). Individual differences in non-verbal number acuity correlate with maths achievement. Nature 455, 665–668. 10.1038/nature07246 18776888

[B15] Harnad S. (1990). The symbol grounding problem. Phys. D Nonlinear Phenom. 42, 335–346 10.1016/0167-2789(90)90087-6

[B16] Jevons W. (1871). The power of numerical discrimination. Nature 3, 281–282 10.1038/003281a0

[B17] Lindskog M. Winman A. Juslin P. (2014). The Association between higher education and approximate number system acuity. Front. Psychol. 5:462. 10.3389/fpsyg.2014.00462 24904478 PMC4033103

[B18] Lindskog M. Winman A. Juslin P. Poom L. (2013). Measuring acuity of the approximate number system reliably and validly: the evaluation of an adaptive test procedure. Front. Psychol. 4:510. 10.3389/fpsyg.2013.00510 23964256 PMC3734355

[B19] Nys J. Ventura P. Fernandes T. Querido L. Leybaert J. Content A. (2013). Does math education modify the approximate number system? A comparison of schooled and unschooled adults. Trends Neurosci. Educ. 2, 13–22 10.1016/j.tine.2013.01.001

[B20] Piazza M. Facoetti A. Trussardi A. N. Berteletti I. Conte S. Lucangeli D.. (2010). Developmental trajectory of number acuity reveals a severe impairment in developmental dyscalculia. Cognition 116, 33–41. 10.1016/j.cognition.2010.03.012 20381023

[B21] Piazza M. Pica P. Izard V. Spelke E. S. Dehaene S. (2013). Education enhances the acuity of the non-verbal approximate number system. Psychol. Sci. 24, 1037–1043. 10.1177/0956797612464057 23625879 PMC4648254

[B22] Rugani R. Fontanari L. Simoni E. Regolin L. Vallortigara G. (2009). Arithmetic in newborn chicks. Proc. Biol. Sci. 276, 2451–2460. 10.1098/rspb.2009.0044 19364746 PMC2690459

[B23] Stoianov I. Zorzi M. (2012). Emergence of a “visual number sense” in hierarchical generative models. Nat. Neurosci. 15, 194–196. 10.1038/nn.2996 22231428

[B24] Stoianov I. Zorzi M. Becker S. Umiltà C. (2002). Associative arithmetic with Boltzmann Machines: the role of number representations, in ICANN, ed Dorronsoro J. (Berlin: Springer), 277–283.

[B25] Stoianov I. Zorzi M. Umilta C. (2003). A connectionist model of simple mental arithmetic, in Proceedings of EuroCogSci03, eds Schmalhofer F. Young R. M. Katz G. (Mahwah, NJ: Lawrence Erlbaum), 313–318.

[B26] Stoianov I. Zorzi M. Umiltà C. (2004). The role of semantic and symbolic representations in arithmetic processing: insights from simulated dyscalculia in a connectionist model. Cortex 40, 192–194. 10.1016/S0010-9452(08)70948-1 15174483

[B27] Viswanathan P. Nieder A. (2013). Neuronal correlates of a visual “sense of number” in primate parietal and prefrontal cortices. Proc. Natl. Acad. Sci. U.S.A. 110, 11187–11192. 10.1073/pnas.1308141110 23776242 PMC3704030

[B28] Zorzi M. Stoianov I. Umiltà C. (2005). Computational modeling of numerical cognition, in Handbook of Mathematical Cognition, ed Campbell J. (New York, NY: Psychology Press), 67–84.

[B29] Zorzi M. Testolin A. Stoianov I. (2013). Modeling language and cognition with deep unsupervised learning: a tutorial overview. Front. Psychol. 4:515. 10.3389/fpsyg.2013.00515 23970869 PMC3747356

